# A Pharyngoplasty with a Dorsal Palatal Flap Expansion: The Evaluation of a Modified Surgical Treatment Method for Obstructive Sleep Apnea Syndrome—A Preliminary Report

**DOI:** 10.3390/jcm10163746

**Published:** 2021-08-23

**Authors:** Ewa Olszewska, Piotr Fiedorczuk, Adam Stróżyński, Agnieszka Polecka, Ewa Roszkowska, B. Tucker Woodson

**Affiliations:** 1 Department of Otolaryngology, Medical University of Bialystok, 15-328 Bialystok, Poland; 2 Doctoral School of the Medical University of Bialystok, 15-328 Bialystok, Poland; piotr.fiedorczuk@umb.edu.pl; 3 Medical University of Bialystok, 15-328 Bialystok, Poland; ad.strozynski@gmail.com (A.S.); polecka.aga@gmail.com (A.P.); 4 Faculty of Economics and Finance, University of Bialystok, 15-062 Bialystok, Poland; e.roszkowska@uwb.edu.pl; 5 Department of Otolaryngology Medical, Division of Sleep Medicine and Upper Airway Reconstructive Surgery, Medical College Wisconsin, Milwaukee, WI 53226, USA; bwoodson@mcw.edu

**Keywords:** obstructive sleep apnea, sleep surgery, pharyngoplasty, dorsal palatal flap expansion

## Abstract

Surgical techniques for obstructive sleep apnea syndrome (OSAS) constantly evolve. This study aims to assess the effectiveness and safety of a new surgical approach for an OSAS pharyngoplasty with a dorsal palatal flap expansion (PDPFEx). A total of 21 participants (mean age 49.9; mean BMI 32.5) underwent a type III sleep study, an endoscopy of the upper airways, a filled medical history, a visual analog scale for snoring loudness, an Epworth Sleepiness Scale, and a Short Form Health Survey-36 questionnaire. A follow-up re-evaluation was performed 11 ± 4.9 months post-operatively. The study group (4 with moderate, 17 with severe OSAS) showed an improvement in all measured sleep study characteristics (*p* < 0.05), apnea-hypopnea index (pre-median 45.7 to 29.3 post-operatively, *p* = 0.009, *r* = 0.394), oxygen desaturation index (pre-median 47.7 and 23.3 post-operatively, *p* = 0.0005, *r* = 0.812), mean oxygen saturation (median 92% pre-operatively and median 94% post-operatively, *p* = 0.0002, *r* = 0.812), lowest oxygen saturation (*p* = 0.0001, *r* = 0.540) and time of sleep spent with blood oxygen saturation less than 90% (*p* = 0.0001, *r* = 0.485). The most commonly reported complications were throat dryness (11 patients) and minor difficulties in swallowing (5 patients transient, 3 patients constant). We conclude that a PDPFEx is a promising new surgical method; however, further controlled studies are needed to demonstrate its safety and efficacy for OSAS treatment in adults.

## 1. Introduction

Obstructive sleep apnea syndrome (OSAS) is a nocturnal disorder of multifactorial causes characterized by recurrent episodes of upper airway obstruction during sleep associated with oxygen desaturation and sleep fragmentation with a variety of methods to diagnose and treat. As a frequent and increasingly prevalent condition associated with serious comorbidities, it poses a great impact on public health [1].

### 1.1. Importance of OSAS

OSAS is a highly prevalent disorder estimated to be 9–38% in the general adult population; 13–33% in men and 6–19% in women [2]. Patients with untreated OSAS are at an increased risk of obesity, hypertension, diabetes mellitus, cardiovascular disease, heart failure, metabolic dysregulation, daytime sleepiness, depression, accidents, strokes, and death [3,4,5,6]. Obesity is also one of the major risk factors for OSAS and there has been a colossal increase in rates of obesity over the past decades around the world; therefore, the prevalence of OSAS could increase further in the coming years [7,8,9]. Therefore, it is important to identify and treat nocturnal breathing disorders early and effectively.

### 1.2. Management Options

The suspicion of OSAS arises from the symptoms of patients, such as snoring and apnea events observed by a bed partner, and frequent arousals assessed by a medical examination and by various questionnaires that measure daytime sleepiness, snoring, and the quality of life. OSAS is diagnosed with a sleep study that measures sleep parameters such as the apnea-hypopnea index (AHI), mean oxygen saturation, and the percentage of time spent with oxygen saturation below specified thresholds [3].

All the treatment methods aim to decrease the obstructive events, to improve the blood oxygen saturation of the patients during sleep, and to enhance the quality of life of both patients and their partners [10].

Treatment of OSAS includes non-surgical and surgical methods. The first-line management is a non-surgical approach involving the education of patients, which is the cornerstone of treatment for any medical condition. This should include a discussion of the pathophysiology, risk factors, and clinical consequences. The patient should be informed of the benefits of weight loss and behavior modification such as avoiding modifiable risk factors (e.g., tobacco smoking, drinking alcohol, sleeping pill administration). Conservative treatment is not successful for a large percentage of patients [11]. The primary treatment modality for OSAS is positive airway pressure therapy (PAP). Alternative or adjunct non-invasive methods that modify the position of the mandible, move the tongue forward, and widen the retrolingual airway, such as oral appliances including mandibular advancement devices, may be offered as per the anatomy, type, and severity of the OSAS of the patient as well as patient preferences. Additional, less established, non-operative management strategies include positional therapy (PT), transcutaneous electrical stimulation (TES), and drug therapy with steroids and leukotriene receptor antagonists [12].

In patients who are not compliant with or fail the PAP therapy, a variety of surgical methods have been used in the past decades. Of those, several surgical approaches are currently being utilized for the most common site of obstruction—the soft palate. Surgical techniques that aim to alter the lateral pharyngeal wall and soft palate at the level of the velopharynx and oropharynx include an expansion sphincter pharyngoplasty, a lateral pharyngoplasty, a relocation pharyngoplasty, a modified uvulopalatopharyngoplasty with uvula preservation, and a suspension palatoplasty [13].

### 1.3. Aim of This Study

The aim of this study is to assess the effectiveness and safety of a new technique of a surgical approach for an OSAS treatment pharyngoplasty with a dorsal palatal flap expansion (PDPFEx).

## 2. Materials and Methods

### 2.1. Ethical Approval and Informed Consent

The study was approved by the local Bioethics Committee. Participation in the study was voluntary. The participants were informed of the study and the data being collected. A written informed consent form (ICF) was obtained from each participant. The participants could withdraw their consent at any point of the study.

### 2.2. Study Design

This was a single-center prospective study with a case series study design for a modification of an expansion sphincter pharyngoplasty described by Puccia and Woodson as a pharyngoplasty with a dorsal palatal flap expansion [14]. 

#### 2.2.1. Study Protocol

The study was conducted at the Department of Otolaryngology in a tertiary care hospital between May 2019 and June 2020. Patients with OSAS were enrolled based on inclusion and exclusion criteria. Surgeries for all cases were performed by the first author (EO).

The inclusion criteria were: (a) adult patients; (b) a moderate or severe OSAS (defined as an apnea-hypopnea index (AHI) of 15–29.9 and ≥30 events/h of sleep); (c) a body mass index (BMI) less than 40; (d) significant snoring with a visual analog scale (VAS) for snoring of 1–10 higher than 8 and (e) a failed PAP therapy.

We excluded patients with severe obesity (BMI ≥ 40), central sleep apnea syndrome, a sleep apnea treatment within the six months preceding the study, previous surgeries involving the hypopharynx and oropharynx areas, a history of rheumatic diseases, a respiratory infection within the previous four weeks, coagulation disorders, chronic or acute kidney failure (defined as serum creatinine > 2.0 mg/dL), the occurrence of other respiratory disorders including chronic obstructive pulmonary disease (diagnosis based on chest radiography and clinical history) or asthma, systemic inflammatory diseases, severe cardiovascular diseases, diabetes, comorbidities affecting systemic inflammation including cancer and collagen vascular disease, chronic rhinosinusitis or receiving medical treatment such as immune suppressors, hormones, free radical scavengers or cytotoxins.

All patients underwent the same procedures according to the study protocol based on a modified SLEEP-GOAL protocol [4]: a medical history, Epworth Sleepiness Scale (ESS) questionnaire, body mass index (BMI), an endoscopy of the upper airways, a sleep study type III polygraph (PG) and a surgical procedure. ESS is a self-conducted questionnaire consisting of eight questions involving the likelihoods of falling asleep in various situations such as sitting and reading, watching TV, sitting inactive in a public place, riding in a car for an hour without a break as a passenger, lying down in the afternoon to rest, sitting and talking to someone, sitting silently after a lunch without alcohol and sitting in a car stopped for a few minutes in traffic [15]. The patient assesses these options on a scale of 0 to 3 (0: never doze; 3: high chance of dozing). The total score can range from 0 to 24. In healthy adults, the normal range of sleepiness differs from 0 to 10. A higher score is associated with increased sleepiness [16]. Patients were asked to fill in the Short Form Health Survey-36 questionnaire (SF-36), which measures the subjective quality of life in both physical and mental health aspects by eight scales: physical functioning (PF), role physical (RP), bodily pain (BP), general health (GH), vitality (VT), social functioning (SF), role emotional (RE) and mental health (MH). Component analyses have shown that there are two distinct concepts measured by the SF-36 questionnaire: a physical dimension represented by the physical component summary (PCS) and a mental dimension represented by the mental component summary (MCS), all summarized as an SF-36 overall score.

#### 2.2.2. Sleep Study

A PG was performed in each case using a type III sleep study device (SOMNOtouch, SOMNOmed). During the PG, the following parameters were evaluated for this study: mean oxygen saturation (MOS) and lowest oxygen saturation (LSAT), time of sleep spent with blood oxygen saturation less than 90% (SpO2 < 90), and the apnea-hypopnea index (AHI).

The AHI is described as the total number of apnea and hypopnea events per hour of sleep recorded in an overnight sleep study. Apneas are defined as at least a 90% decrease in airflow for at least 10 s and hypopneas as reduction of respiratory signals for at least 10 s associated with a minimum of 3% of oxygen desaturation [17]. MOS is estimated as normal varies between 94% and 98% during sleep [3].

### 2.3. Description of the Surgical Procedure

**Figure 1:**
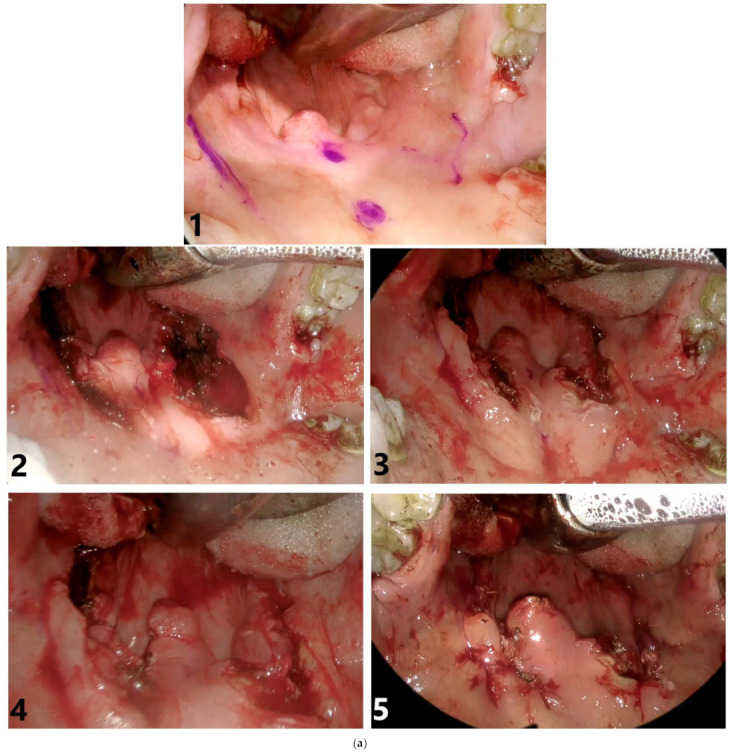

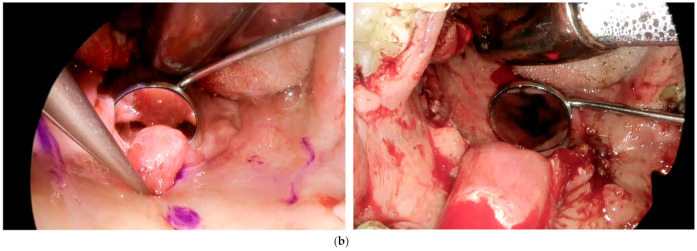
(**a**) Endoscopic view. 1: Surgery site with markings at the left and right pterygomandibular raphes, the base of the uvula and the hard palate; 2: palatal tonsils removed and ventral palate triangles of mucosa removed; 3: supratonsillar fat tissue removed and palatopharyngeus muscle dissected from the superior constrictor muscle; 4: dorsal palatal flaps rotated; left flap sutured in place and right flap prepared for suturing and 5: bilateral suturing of the dorsal flaps. (**b**) On the left: the retropalatal space before the surgery and the assessment of the length of the uvula for excising the tip. On the right: a significantly increased retropalatal space after the surgery and a view after removal of the enlarged uvula.

The surgical procedure was a further modification of an expansion sphincter pharyngoplasty, which was previously developed by Pang and Woodson to mitigate a fairly low success rate with a traditional uvulopalatopharyngoplasty in OSAS patients [18]. The current modification, hereby referred to as a pharyngoplasty with a dorsal palatal flap expansion (PDPFEx), was first described in print in 2020 by Puccia and Woodson (Figure 1) [14].

The surgical steps of a PDPFEx are: (1) the removal of the triangle (base of the triangle at the free edge of the palate on both sides of the uvula with the apex of the triangle extending anteriorly approaching the hard palate) of mucosa at the ventral palate to expose the surgical field; (2) the removal of supratonsillar fat tissue; (3) the dissection, incision and separation of the palatoglossal muscle and to expose the palatopharyngeus muscle; (4) dissection of the freeing of the palatopharyngeus muscle from the superior constrictor muscle; (5) a vertical incision on the dorsal palatal mucosa immediately lateral to the uvula and (6) the anterolateral rotation of this dorsal palatal mucosal flap, suturing it to the lateral edge of the ventral palatal mucosa.

### 2.4. Study Assessments

After surgery, the subjects were invited to a follow-up visit 4–6 months after the surgery. During the visits, the patients were examined by a medical professional and asked about complications regarding the post-surgery period, given the modified SLEEP-GOAL protocol (patient data, blood pressure, ESS score, snoring VAS, comorbidities, ENT examination results, SF-36 questionnaire). A post-surgery type III sleep study was given to the patients to obtain the sleep parameters.

### 2.5. Post-Operative Patient Care

The patients were closely monitored in hospital conditions for a median of 2 days after the procedure and were administered intravenous pain medications (paracetamol and metamizole; tramadol when the reported pain VAS was 6 or higher) and dexamethasone (to decrease the soft tissue edema). After they were discharged, the patients were prescribed all three pain medications to be taken orally for as long as the pain persisted.

### 2.6. Study Outcomes

The primary study outcomes consisted of differences in the AHI, ESS, and blood oxygen saturation characteristics established in sleep studies at the baseline and the follow-up.

### 2.7. Statistical Analysis 

The statistical analysis was performed using GraphPad Prism 9. The results are presented as mean ± SD or a number of patients and a percentage of the whole study group. The categorical variables are presented as percentages. For a comparison of the results, the Wilcoxon signed-rank test was used. *p*-values < 0.05 were accepted as statistically significant.

## 3. Results

### 3.1. Study Group

A total of 21 patients (adult men) with an average age of 49.9 ± 12.2 (range 26–72 years old) were included in the study between May 2019 and June 2020. By polygraphy, 4 moderate OSAS (15 ≤ AHI ˂ 30) and 17 severe OSAS (AHI ≥ 30) patients were identified. The biometrics of the patients (including the mean age (49.9 ± 12.2), mean weight (101.9 ± 10.2), and mean BMI (32.5 ± 3.4)) were taken; the blood pressure was measured and the mean arterial blood pressure was 105.4 ± 8.4. The MAP was calculated using the formula (Systolic+2(Diastolic)/:3. A total of 15 patients had existing comorbidities and cardiovascular complications including 5 patients with more than one comorbidity. The palatal anatomy of patients was assessed during an otorhinolaryngological examination; the Friedman Tongue Scale and the Friedman Palatine Tonsils Scale were used. Most of the patients were classified as Friedman Tongue position 3. The patients were asked to fill in ESS and snoring VAS questionnaires. The baseline group characteristics are provided in Table 1.

### 3.2. Study Outcomes

The follow-up visits were held between May 2020 and December 2020, with a follow-up period that ranged from 4 to 21 months (median 11 months). All 21 patients completed their follow-up; however, the planned 4–6 month period was elongated by the COVID-19 global pandemic. The biometric characteristics of the study group (body weight, BMI, mean arterial blood pressure) did not change during the post-operative period. (Table 2).

**Figure 2:**
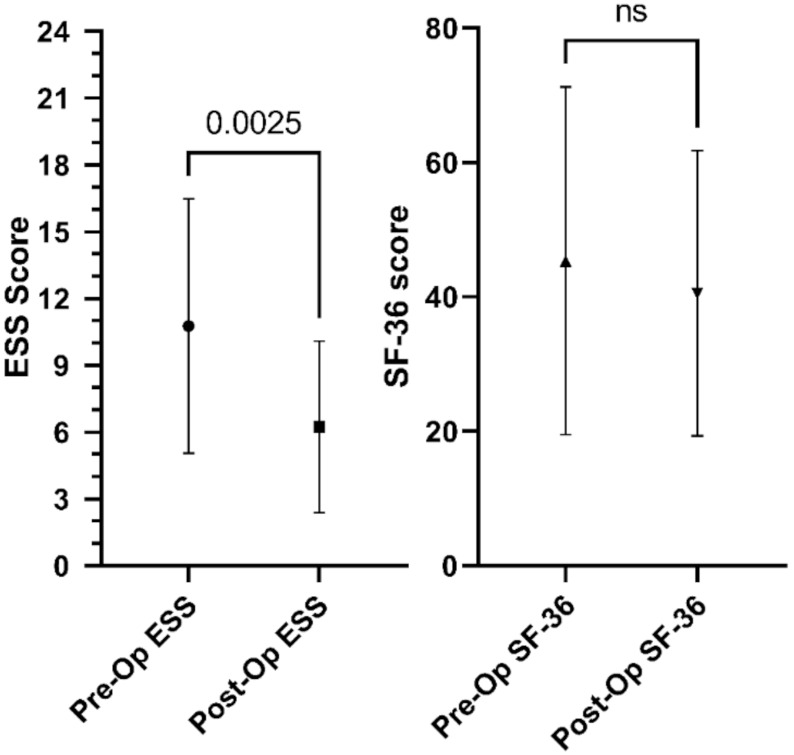
Pre- and post-operative comparison of the Epworth Sleepiness Scale and the SF-36 overall score. The exact *p*-value is shown above the columns.

There was an improvement in the subjective daytime sleepiness of patients as ranked by the ESS questionnaire score; there was a change in the ESS from a median of 9.0 (IQR 7–17) to 7.0 (IQR 2.5–9.5). No significant difference was observed in the self-reported quality of life assessed by the SF-36 questionnaire in any of the subscales, in the physical and mental component summary nor the SF-36 overall score (Figure 2).

**Figure 3:**
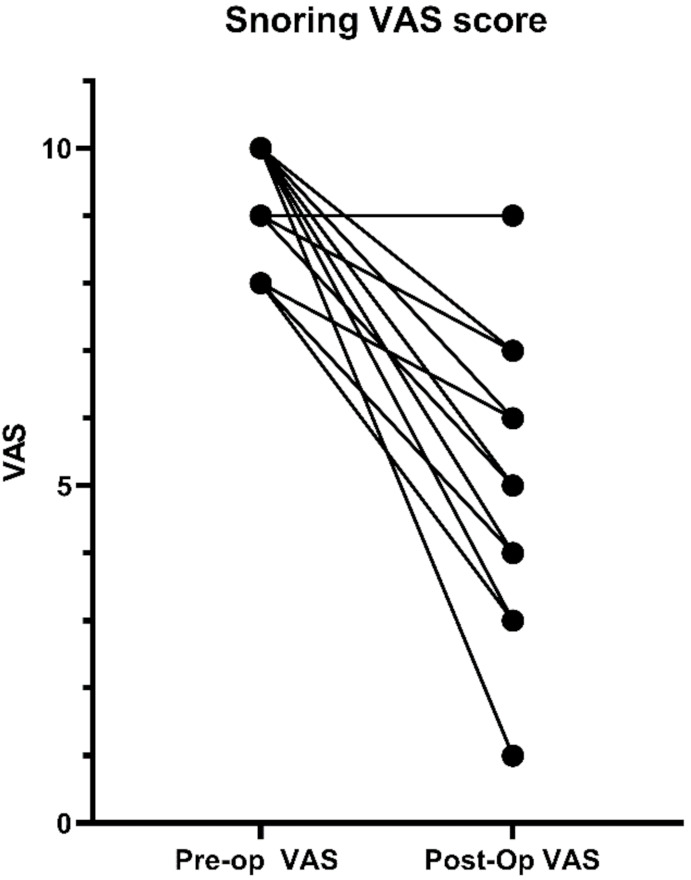
Pre- and post-operative comparison of the reported visual analog scale score for snoring loudness and inconvenience by the patients.

Most patients in our study group reported a decrease in snoring loudness and inconvenience assessed with the snoring visual analog scale. The snoring VAS changed from a median of 10 (IQR 8.5–10) to 5 (IQR 4–6) (Figure 3).

**Figure 4:**
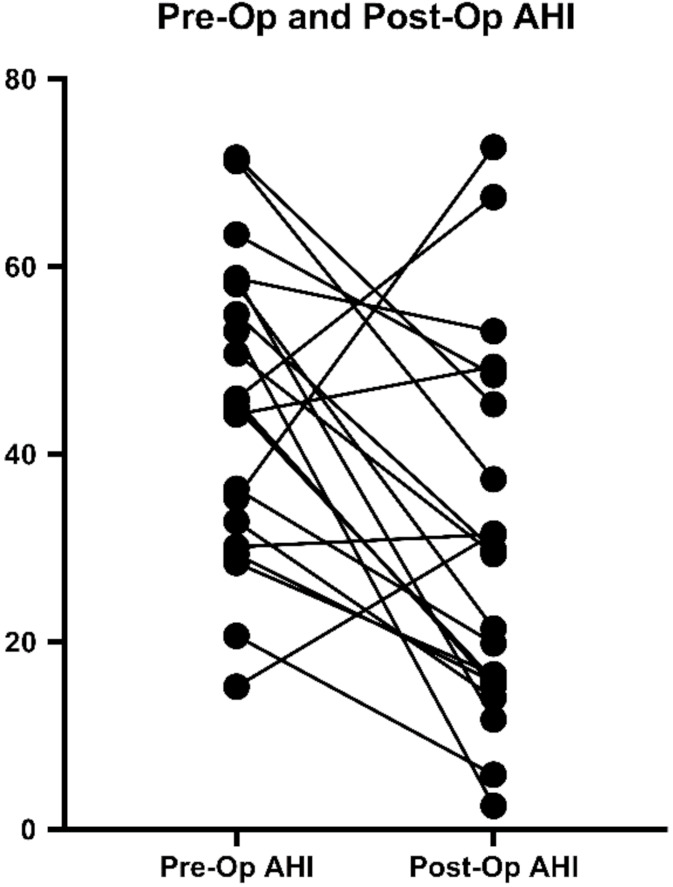
Individual pre- and post-operative AHI distribution. AHI: apnea-hypopnea index.

**Figure 5:**
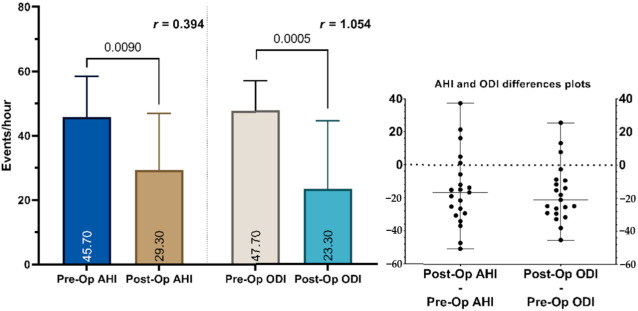
On the left side: a pre- and post-operative comparison of the apnea-hypopnea index and the oxygen desaturation index median and interquartile range values. The median values are shown inside the columns and the *p*-value is shown above the columns. The effect size (*r*-value) is shown above the columns. On the right side: differences plot for the AHI and ODI of individual patients. AHI: apnea-hypopnea index; ODI: oxygen desaturation index.

All subjects had a post-operative PG as well. When comparing the pre-operative and post-operative PG characteristics, we observed significant improvements in the AHI from a median of 45.7 (IQR 31.5–58.4) to 29.3 (IQR 15.5–46.9) and in the ODI from a median of 47.7 (IQR 34.7–57.1) to 23.3 (IQR 12.5–44.4) (Figure 4 and Figure 5). There was a decrease in the AHI of 76.1% and a decrease in the ODI in 85.7% of the patients in the study group.

**Figure 6:**
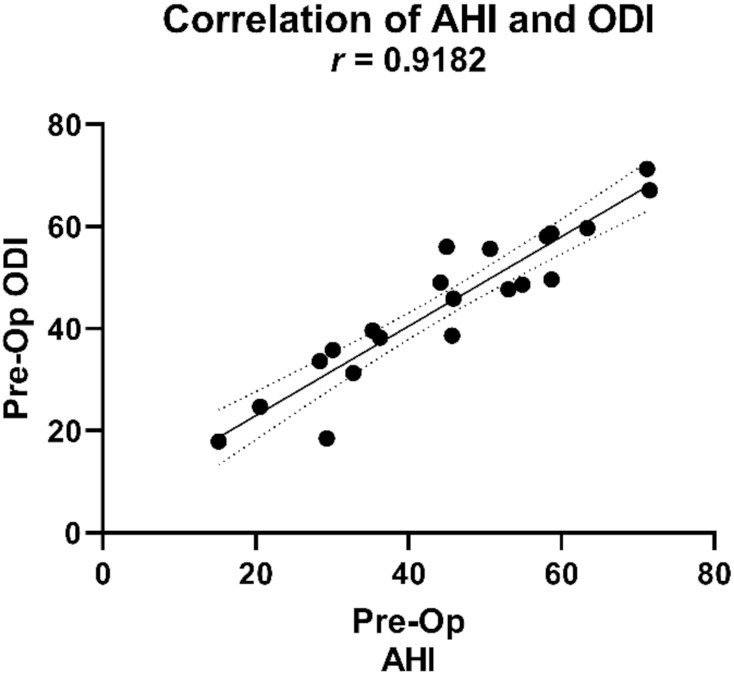
Correlation between the pre-operative AHI and ODI. AHI: apnea–hypopnea index; ODI: oxygen desaturation index; *r*: Spearman’s rank correlation coefficient.

Our results showed a lower median of the pre-operative ODI than the pre-operative AHI (45.7 and 47.7, respectively) yet a Wilcoxon signed-rank test gave no statistical difference between the compared values (*p* = 0.8986). We observed a significant correlation between the two variables, as shown in Figure 6.

**Figure 7:**
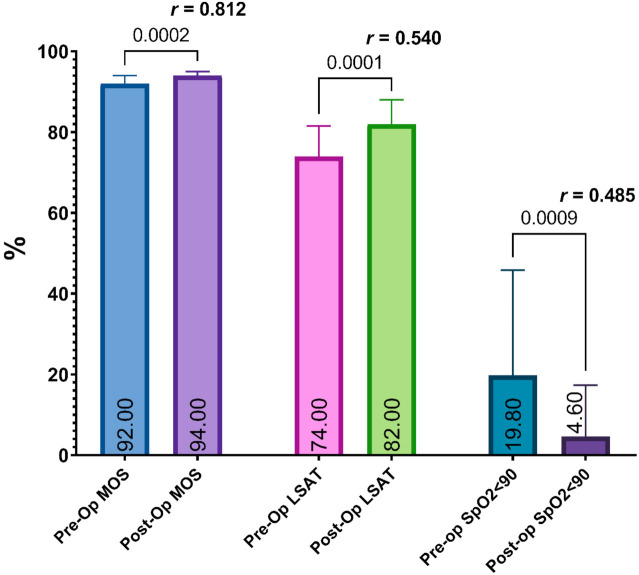
A pre- and post-operative comparison of the median and interquartile range values of the mean oxygen saturation, lowest oxygen saturation, and time of sleep spent with blood oxygen saturation less than 90%. The median values are shown inside the columns and the *p*-value is shown above the columns. The effect size (*r*-value) is shown above the columns.

Additionally, we observed an improvement in the MOS from a median of 92.0 (IQR 90.3–94.0) to 94.0 (IQR 93.0–95.0), the LSAT from a median of 74.0 (IQR 68.5–81.5) to 82.0 (IQR 74.0–88.0) and the SpO_2_ < 90% from a median of 19.8 (IQR 5.9–45.8) to 4.6 (IQR 0.3–17.3) (Figure 7).

### 3.3. Post-Operative Complications

After the hospital stay, patients were asked about post-operative pain (duration of pain in days, pain level described on the VAS scale, painkiller intake with a total of three recommended drugs and usage duration) and other symptoms such as throat dryness (prevalence, time of symptom onset and duration), palate hematoma (prevalence, time of symptom onset and duration), post-operative secondary hemorrhage (early (less than 10 days post-operative) and late (10 days or more post-operative)), difficulty in swallowing (prevalence, time of symptom onset and duration), impaired taste (prevalence, time of symptom onset and duration) and other post-operative remarks.

The post-operative pain persisted for a median of 14 days (IQR 10–21) with a median VAS of 8 (IQR 6–9). A total of 18 patients took all 3 medicaments and for 3 patients the pain was manageable without the opioid. The median drug use duration of the patients was a total of 14 days (IQR 14–21). In 63.2% of cases, the pain lasted for less than 14 days.

**Figure 8:**
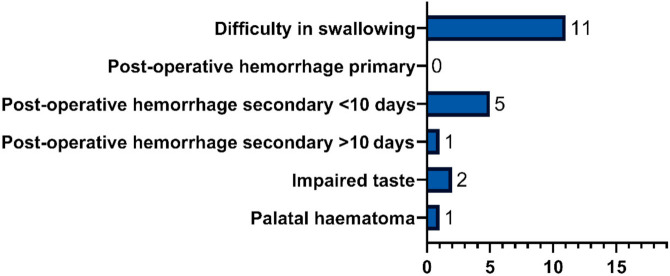
Post-operative symptom occurrence graph showing the number of patients with a post-operative complication.

The most commonly reported complications were throat dryness and minor difficulties in swallowing. Figure 8 shows a graph of the occurrence of reported complications.

Throat dryness was reported by 11 patients. In 9 cases, the symptom appeared immediately after the surgery, and 2 patients noticed the dryness a few days after. A total of 7 patients complained about the symptom for more than three months, 1 up to three months, 2 up to three weeks, and in 1 case the dryness lasted three days. Only 1 patient reported a palatal hematoma that appeared two days after the surgery and persisted for two weeks. No primary post-operative hemorrhage was observed but secondary bleeding occurred in 6 cases. A total of 8 patients described a difficulty in swallowing that appeared immediately after the surgery. Of those, 3 patients reported the difficulty for more than three months after; in 2 patients it lasted up to two months, 1 up to one month, and in 2 cases it lasted for two weeks. An impaired taste was noted by 2 patients and, in both cases, it lasted for more than three months. Other mentioned symptoms included a sense of thick mucus in the throat (3 cases), tongue numbness (2 cases), small amounts of blood in nasal discharge (1 case), cough (1 case), a sense of foreign body in the throat (1 case) and a gag reflex (1 case). No major post-operative complications, e.g., major bleeding or palatopharyngeal insufficiency, were noted.

## 4. Discussion

The goal for all pharyngoplasties is to widen the retropalatal airspace in both the anterior-posterior dimension and between the lateral pharyngeal walls and stiffen the tissues. Interrupting and re-directing the sphincteric action of palatopharyngeal muscles contributes to the widening of the space between the palate and the posterior pharyngeal wall and the lateral dimension of the pharynx. A pharyngoplasty with a dorsal palatal flap expansion has distinct steps that are contributory to each other. Each step is necessary for and facilitates the next. The removal of the anterior triangle of mucosa exposes the surgical field to remove the supratonsillar fat tissue, which exposes the palatoglossus and palatopharyngeus muscles and facilitates their dissection and manipulation. The removal and relocation of these intravelar tissues expose the mirror image of the anterior palatal mucosal triangle at the dorsal palatal mucosa. This dorsal palatal mucosal triangle is freed up by cutting at the medial limit to freely rotate anterolaterally along the axis of the lateral edge of this triangle to cover the lateral palatal space.

This new modification (PDPFEx) has distinct differences compared to the previous technique (ESP): (1) there is enhanced dissection and mobilization of the palatopharyngeus muscle to facilitate (provide space) for the creation and rotation of the dorsal palatal flap; (2) the preservation of the mucosa of the nasopharyngeal surface of the soft palate; (3) the use of this mucosa to cover the anterolateral raw surgical dissection field; (4) the shortening of the medial portion of the soft palate lateral to the uvula resulting in an increased anteroposterior dimension of the nasopharynx; (5) the repositioning of the uvula slightly more anteriorly; (6) the shortening of the elongated uvula due to the medial cuts for the dorsal palatal flap; (7) as a result of these same cuts lateral to the uvula that form the dorsal palatal flap, there is a reduction of the sphincter function of the transverse bundle of the palatopharyngeus muscle.

The success of pharyngoplasties depends on an accurate diagnostic assessment, good patient selection, a well-performed surgical technique, and an uncomplicated post-operative period. Here, we presented our experience with the new modification of the expansion sphincter pharyngoplasty technique for OSA treatment described by Woodson [14]. Having followed the described surgical protocol, we assessed the effectiveness and reported the promising outcomes as improvements in sleep parameters such as the AHI, ODI, MOS, LOS, and T < 90%. This correlation is consistent with the available literature, e.g., as reported by Hussain Basheer et al. who reported a similarly strong correlation between the ODI and AHI [19]. In all assessed sleep parameters, a significant improvement was observed at the final follow-up visit. Although our results were generally similar to those obtained by other authors, we observed a higher degree of improvement compared to the literature in several parameters such as the change in the percentage of lowest oxygen saturation post-operatively [10,20]. Our study may be limited by the number of patients; however, it was adequately powered to establish efficacy and statistical differences. On the other hand, this could partly be a sign of the “regression to the mean” or by random due to the small study sample. Plaza et al. conducted a prospective non-comparative multicenter study of patients who underwent an expansion sphincter pharyngoplasty procedure as a treatment for OSAS and showed a significant improvement in the AHI and ESS post-operatively; however, other sleep parameters were not presented [21]. El-Ahl and El-Anwar described that the lowest oxygen saturation increased significantly in a group of 24 patients with Friedman stage II and III of OSAS [22], which was consistent with our study. In all three publications mentioned above, there was no statistical difference between BMI before and after the surgery, which was similar to our results [10,20,21].

In our study, we compared the mean arterial pressure before and at the final follow-up visit and observed no change. Blood pressure also remained unchanged in the MacKay study [20]. Another parameter evaluated in our study group was the snoring loudness that changed after the surgical treatment. The VAS scale for snoring loudness showed a statistically significant difference. It may mean that this modified technique results in a decrease in pharyngeal tissue vibration.

Our results showed a relatively high percentage of pain on day 14 post-operatively. However, this was in part due to the patients who did not follow the pain management protocol strictly. Moreover, improved sleep parameters and favorable patient satisfaction outcomes may justify the relatively prolonged pain experience.

Pang et al. in their meta-analysis on the effectiveness of palatal surgery showed the superiority of more innovative, anatomically targeted surgical procedures over a traditional uvulopalatopharyngoplasty [23]. This conclusion was consistent with the research conducted by El-Ahl and El-Anwar [22]. Other studies have also shown good results after expansion techniques as a treatment for OSA but more information is needed to modify and improve the sleep surgery methods [24].

Although this current study was not controlled, comparing it to other techniques, we are in the process of expanding our series and designing future controlled studies with additional pre-and post-operative assessments to test the effectiveness of this technique. When the COVID-19 pandemic started in March 2020, several patients refused to submit to a post-operative follow-up in the scheduled time, which further increased the mean group follow-up period. Second, this study included a select population of individuals of white European origin only. Third, primal differences in the characteristics among patients—especially age, with the youngest participant at age 27—could affect the regeneration process and thus have an impact on the progress. Additionally, women were underrepresented in the trial. Therefore, the generalization of findings to sex, populations with a low prevalence of obesity, or people from other ethnic backgrounds was limited. Although the study was limited by the number of patients, it did establish efficacy and statistical differences in several variables. Another limitation involved the diagnosis of OSAS. In this case, we used study type III. We are aware of the limitations that this type of sleep study creates in quantifying the AHI and identifying arousals and in its inability to assess the sleep structure. However, in all participants, we conducted two-night sleep studies, which have advantages compared to one-night polysomnography (PSG). The first-night effect of the sleep study may be a limitation in the single-night PSGs. To reduce the negative impact of home sleep studies, the participants enrolled in our study were thoroughly instructed on how to put them on and how to use the device. Any individuals with comorbidities were excluded from the study. The Academy of Sleep Apnea approves the performance of a sleep study type III when the patient is free from significant comorbid conditions and for a pre-operative clinical evaluation [3,25]. Other studies have indicated that a home portable monitoring device showed a high level of diagnostic agreement between the home diagnostic sleep studies and a simultaneous PSG, especially when manually scored [26]. The difference between the home study and the AHI from the reference PSG as demonstrated by the authors was similar to the difference between the PSGs. Bibbins-Domingo et al. demonstrate that a home sleep study is recommended for the diagnosis of OSAS in uncomplicated patients with an increased risk of moderate-to-severe OSAS as a viable alternative to a PSG in selected circumstances [27]. In the current study, the sleep study was read manually by the first author.

Based on our previous experience and outcomes, we believe that the technique allowed for better control of the muscle tension and sphincteric activity of the palatopharyngeal muscle. Single-arm studies on a specific surgical technique may show better outcomes in a few variables compared to other techniques [18]. However, many potential variables related to the subject characteristics in these studies avoid the comparison of the outcomes between the techniques to draw conclusions. Randomized clinical trials that stratify the important subject factors facilitate such comparisons [10,28,29]. More studies are needed with larger sample sizes and control groups to better demonstrate the differences between the expansion techniques as well as to compare the effectiveness of a PDPFEx and non-surgical treatment for OSA. Preliminary case series such as this may provide outcome data that may serve to assess the magnitude of the effect and the sample size calculation for a randomized clinical trial. On the other hand, comparing a PDPFEx to another technique with a similarly favorable outcome may not be feasible due to the required large sample size.

## 5. Conclusions

A better understanding of the palatal anatomy has been applied to reconstructive palatal surgery for the treatment of OSAS. The palatopharyngeus muscle is a major defining element of the palate and lateral pharyngeal wall. This muscle is the key to many current reconstructive pharyngoplasty techniques. This prospective single-center work on a new modification of a pharyngoplasty—a pharyngoplasty with dorsal palatal flap expansion—shows that this technique can provide good outcomes in patients with OSAS. This modification in the technique appears to have advantages in not only changing palatal muscle vectors and reducing the mass of tissues that vibrate and/or collapse but also by expanding the retropalatal space. However, this presumed mechanism remains speculative in the absence of objective measurements such as an MRI or DISE. Further studies should be conducted to adequately compare a PDPFEx with other surgical approaches regarding an improvement in sleep study characteristics and an increase in the quality of life and safety of patients. 

## Figures and Tables

**Table 1:** Baseline characteristics of the study group (*n* = 21 male subjects).

Characteristic	Mean (SD)/*n*%	Min	Max
**Biometrics**			
Age (y)	49.9 (12.2)	26	72
Weight (kg)	101.9 (10.2)	85	120
Body Mass Index (kg/m^2^)	32.5 (3.4)	26.3	39.7
BMI group I *	6–28.6%		
BMI group II *	10–47.6%		
BMI group III *	5–23.8%		
Mean Blood Pressure (mmHg)	105.4 (8.4)	90.7	121.3
**Comorbidities**	15–71.4%		
Hypertension	15–71.4%		
Diabetes Mellitus Type 2	4–19%		
Heart Disease	2–9.5%		
**Anatomy and OSAS**			
Friedman Tongue Position		2	4
Friedman Tongue Position 2	3–14.3%		
Friedman Tongue Position 3	16–76.2%		
Friedman Tongue Position 4	2–9.5%		
Friedman Palatine Tonsils Scale		1	3
Friedman Palatine Tonsils Scale 1	9–42.9%		
Friedman Palatine Tonsils Scale 2	7–33.3%		
Friedman Palatine Tonsils Scale 3	5–23.8%		
Epworth Sleepiness Scale	10.8 (5.7)	1	22
Apnea–Hypopnea Index	45.2 (15.9)	15.2	71.6
Moderate OSAS (15 ≤ AHI ˂ 30)	4–19%		
Severe OSAS (AHI > 30)	17–81%		
Snoring Visual Analog Scale	9.4 (0.9)	8	10

* BMI range in this study: group I (normal weight or overweight) 18.5 to 29.9, group II (class I obesity) 30 to 34.9, group III (class II obesity) 35 to 39.9. Heart disease included arrhythmias, heart failure, heart valve disease, cardiomyopathy, and congenital heart disease.

**Table 2:** Study outcome results.

	Median Pre (IQR)	Median Post (IQR)	Significance	Effect Size *r*
**Biometric Characteristics**				
Weight	100.0 (92–111.5)	100.0 (93.5–109)	0.275	0.168
BMI	32.6 (29.7–34.8)	32.3 (30.1–34.1)	0.278	0.167
MAP	103.7 (98–111.4)	101.7 (93–109.7)	0.251	0.177
**Questionnaires**				
Snoring VAS	10 (8.5–10)	5 (4–6)	<0.0001	0.738
ESS	9.0 (7–17)	7.0 (2.5–9.5)	0.0025	0.442
SF-36	41.0 (27.5–58)	35.0 (19–53.3)	0.715	0.056

IQR: interquartile range; BMI: body mass index; MAP: mean arterial blood pressure; VAS: visual analog scale; ESS: Epworth Sleepiness Scale; SF-36: the Short Form Health Survey-36. The results were compared using the Wilcoxon signed-rank test.

## Data Availability

The data presented in this study are available on request from the corresponding author. The data are not publicly available.
